# Functional Importance of Surface Texture Parameters

**DOI:** 10.3390/ma14185326

**Published:** 2021-09-15

**Authors:** Pawel Pawlus, Rafal Reizer, Michal Wieczorowski

**Affiliations:** 1 Faculty of Mechanical Engineering and Aeronautics, Rzeszow University of Technology, Powstancow Warszawy 8 Street, 35-959 Rzeszow, Poland; ppawlus@prz.edu.pl; 2 College of Natural Sciences, University of Rzeszow, Pigonia Street 1, 35-310 Rzeszow, Poland; rreizer@ur.edu.pl; 3 Division of Metrology and Measurement Systems, Faculty of Mechanical Engineering, Institute of Mechanical Technology, Poznan University of Technology, Piotrowo Street 3, 60-965 Poznan, Poland

**Keywords:** surface texture, parameters, functional importance

## Abstract

Areal 3D analysis of surface texture gives more opportunities than a study of 2D profiles. Surface topography evaluation, considered as 3D dimensional analysis in micro or nanoscales, plays an important role in many fields of science and life. Among many texture parameters, those connected with height are the most often used. However, there are many other parameters and functions that can provide additional important information regarding functional behaviour of surfaces in different applications. The knowledge about the functional importance of various surface properties is low. This review tries to fill this gap. Surface texture parameters are presented in various groups: height, spatial, hybrid, functional, feature, and others. Based on experiences of the present authors and literature review, the relationships among various surface parameters and functional properties are described. A proposal of a selection of parameters on the basis of their functional significations is provided. Considerations for future challenges are addressed.

## 1. Introduction

Surface topography evaluation is functionally important. Some properties, such as those of material in contact, sealing, friction, lubricant retention, and wear resistance, are related to the surface topography. Surface topography is the fingerprint of manufacturing processes, but also of functioning conditions and wear processes. Therefore, the results of surface topography measurement are important for manufacturers and tribologists. The first profilometer was constructed by Abbott et al. [1]. Abbott and Firestone published one of the first papers related to description of the roughness profile. They defined a material ratio curve [2], important in tribology. The material ratio of the profile is the ratio of the sum of the profile elements at a given level to the evaluation length. There are various types of roughness parameters, such as height, spacing, and hybrid [3]. The number of profile roughness parameters increased [4] and some parameters were correlated with others. However, the surface topography is three-dimensional in nature. Three-dimensional (areal) surface parameters are more reliable than 2D profile parameters; for example, a peak on a 2D profile is not necessarily a summit on areal surface topography, especially for random surfaces. The mean gradient of the surface is larger than the mean slope of the profile. Nayak [5] developed relations between statistics of the 2D random profile and 3D Gaussian surfaces, one the basis of works of Longuet-Higgins on ocean surfaces [6,7]; however, their usefulness is limited. Often, a set of profiles will supply an adequate description of surface profiles [8].

Measurement of areal surface texture started in the early 1980s. Somicronic, a small company near Lyon in France, delivered a prototype 3D stylus system to the Ecole Centrale de Lyon in 1990. Somicronic was also the first manufacturer to introduce a wide range of parameters into its software in 1994 [9].

The great major step forward for the development of characterisation in the three dimensions came in 1990 when the European Community supported a research contract awarded to the University of Birmingham and Ecole Centrale de Lyon. The result of this research was a report that proposed the so-called “Birmingham 14” parameters. The number of parameters was restricted to avoid the existence of too many parameters, as in roughness profiles analysis [4]. Next, the SURFSTAND project was born. The results of the SURFSTAND project form the basis of the books [9,10]. However, in the new ISO 25178-2 [11] standard many parameters exist. They were described in [12].

There are many areal parameters in ISO 25178-2 standard. It is not necessary to analyse all of them. Some of them describe similar surface properties. Information about all of them may be redundant. Therefore, the number of them should be restricted. The analysis of the correlation between parameters is helpful in their choice. Anderberg et al. [13] studied the correlation of parameters of honed surfaces.

**Figure 1:**
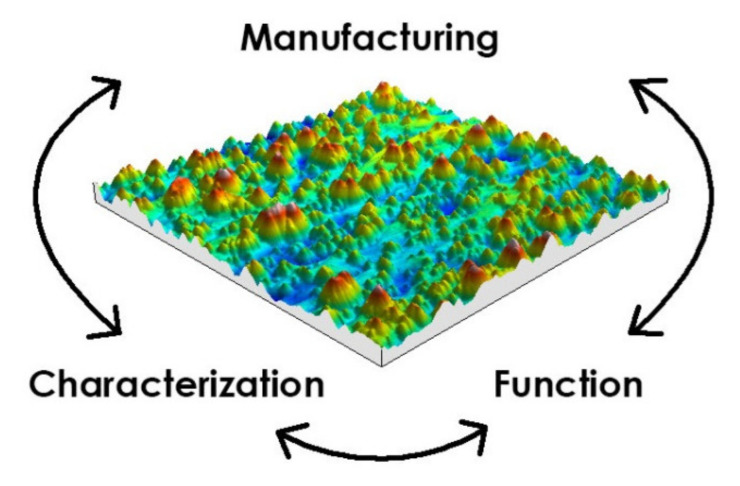
Connections of surface texture.

On the other side, in industry, Ra parameter is often used. However, it is not possible to describe the surface after two processes (such as the plateau-honed cylinder surface) using only it (or its areal version—Sa). The selected parameters should be function-relevant [14,15] and manufacture-related (Figure 1). They can be determined easily.

However, only height parameters are typically used. Other parameters, such as spatial, hybrid or functional, can provide more information on surface texture. The knowledge about the functional importance of various surface properties is low. It is difficult to find a comprehensive review on this topic. In this paper, the authors will present the definitions of areal surface texture parameters with their applications. Especially, parameters included in ISO 25178-2 standard will be analysed.

The field parameters are calculated for all points on the analysed surface. They include height, spatial, hybrid, and other parameters (functional and fractal).

## 2. Height Parameters

The *Sq* parameter is a root mean square (rms) value of surface amplitudes [11].

(1) $$Sq=\sqrt{\frac{1}{A}\iint_{A}z^{2}\left(x,y\right)dxdy},$$

where: *A*—the definition area; *z*—surface height in position *x*, *y*; *x*, *y*—lengths in perpendicular directions. *Sa* is the arithmetical mean of the absolute surface heights.

(2) $$Sa=\frac{1}{A}\iint_{A}^{}\left|z(x,\;y\right|dx\;dy),$$

Skewness *Ssk* is the ratio of the average cube value of the surface ordinates to the cube of the *Sq* parameter [11].

(3) $$Ssk=\frac{1}{\;Sq^{3}}\left[\frac{1}{A}\iint_{A}z^{3}\left(x,y\right)dxdy\right],$$

Kurtosis *Sku* is the ratio of the average quartic value of the surface ordinates to the fourth power of the *Sq* parameter [11].

(4) $$Sku=\frac{1}{Sq^{4}}\left[\frac{1}{A}\iint_{A}z^{4}\left(x,y\right)dxdy\right],$$

*Sp* is the maximum peak height and *Sv* is the maximum valley depth. Maximum height of surface *Sz* (also named *St*) is the sum of the maximum peak height *Sp* and maximum valley depth *Sv* [11].

These parameters are natural extensions of the roughness height parameters. The parameters *Sq, Sa, Sp, Sv*, and *Sz* characterise the surface amplitude, while *Ssk* and *Sku* describe the character of the height distribution. The opinion exists that the maximum height is related to surface damage while the averaged parameters are related to surface normal functioning [15]. The earliest profilometers determined the maximum roughness height [16]. In 2D profile analysis, the *Ra* parameter (arithmetical mean of profile deviation from the mean line) was the most popular, followed by *Rz* (ten-point height) and *Rt* (maximum height). There were many amplitude parameters to characterise the roughness profiles, such as *R3z* or *Rtm*, they were typically developed for characterisation of cylinder liner surfaces [17,18]. The *Ra* parameter is still popular in manufacturing industry [19]. Typically, surface height is minimised in machining.

**Figure 2:**
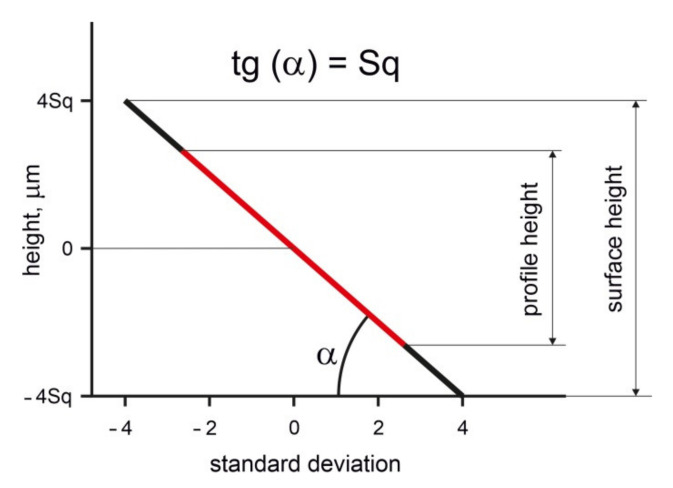
Difference between 3D and 2D heights of the random Gaussian surface.

The parameters *Sq* and *Sa* are similar (typically a little higher) to the roughness parameters *Rq* and *Ra,* respectively, of isotropic surfaces. The isotropic surface has similar profiles in various directions, in contrast to the anisotropic surface. The *Sq* and *Sa* parameters of the anisotropic one-directional surface are similar to the *Pq* and *Pa* parameters (a root mean square value of the profile amplitudes and arithmetical mean of the absolute profile heights, respectively) of the surface measured across the lay (main direction). However, Ohlsson et al. [20], after analysis of honed textures, and Tsukada and Kanada [21] and Wieczorowski et al. [22], after analysis of ground and lapped surfaces, found that the parameters that characterised the maximum height of random surfaces were much higher than the corresponding parameters of the profile. For example, Tsukada and Kanada obtained the ratio of maximum heights in 2D and 3D systems of ground surfaces near 1.8. The difference between 3D and 2D maximum height parameters depends on the number of measured points and the correlation between surface ordinates. The older standard [9] among the averaged amplitude parameters contained only the *Sq* parameter because of its statistical character. A model proposed by Nayak [5] for random surface description using spectral moments was commonly used. The zeroth moment m0 is the variance of the surface height, which is square of the *Pq* parameter *Sq (Pq)* or *Sq* parameter of the isotropic surface. The *Sq(Pq)* parameter can be calculated on the basis of the probability plot of cumulative height distribution of Gaussian surface—Figure 2. Profile height of the Gaussian surface is similar to the *Pq* parameter magnified by three. When the areal (3D) height is restricted to the material ratio in the range: 0.13–99.87%, maximum surface height is similar to the profile height.

Some optical methods, such as scattering, allow one to determine the *Sq* parameter [23]. The random Gaussian surface is modeled on the basis of the *Sq* parameter [24,25,26,27,28]. Wu [24] modified the surface model of surface with Gaussian probability distribution and assumed main wavelength. This method behaved better for larger wavelengths than that developed by Hu and Tonder [25]. You and Ehmann [26] used time series models and Fourier transform. In the recent model developed by Pérez-Ràfols and Almqvist [27], the power spectrum and ordinate distribution can be independently specified. Pawlus et al. [28] compared various methods of random surface modelling. The standard deviation of the surface height was used in tribological investigations to compare tribological behaviour of one-process and two-process surfaces by Jeng [29] and Grabon et al. [30]. The *Sq* parameter is connected to the standard deviation of summit heights, important in contact mechanics [31,32,33,34,35]. Greenwood and Tripp [31] developed a statistical elastic model, while Chang et al. [32], Zhao et al. [33], Kogut and Etsion [34], and Jackson and Green [35] developed elasto-plastic models of the contact between rough surfaces. The deviation between the standard deviations of summit heights and of surface heights depends on the correlation between data points [36]. According to Greenwood and Tripp [31], the contact of two rough textures can be substituted by the contact of the smooth flat and the equivalent texture. Standard deviation of the equivalent surface is equal to the sum of standard deviations of individual textures [37]. However, the *Sa* parameter as the extension of the *Ra* parameter, used frequently in industry, is still popular in research works in the fields of tribology [38] and machining [39,40], therefore the *Sa* parameter was included in ISO 25178-2 standard. Smaller values of the *Sa* and *Sq* parameters correspond to higher surface glossiness [41].

**Figure 3:**
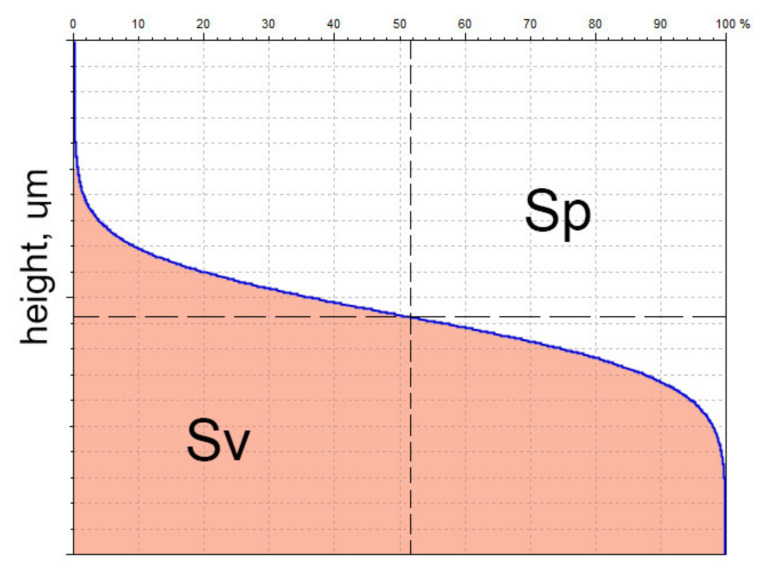
Interpretations of the *Sp* and *Sv* parameters.

There are important interpretations of the *Sp* and *Sv* parameters, similar to the *Rp* and *Rv* parameters [42]. *Sp* means void volume, while *Sv* means material volume (Figure 3).

This interpretation is important, since on the basis of change in the *Sv* parameter of relocated surfaces before and after the tribological test, one can obtain information if wear removal or plastic deformation occurred. When the change of *Sv* is similar to 0, plastic deformation occurs [43]. On the basis of this interpretation, *Sp/Sz* (or *Rp/Rz*) is called the emptiness coefficient [44]. The maximum wear *Sz* can be used for detecting outliers.

During low wear (smaller than a maximum height of initial surface texture) typically the height decreased. The decreases in the values of the *Sp/Rp* parameter are the highest, while the changes in the value of are the *Sv/Rv* parameter the smallest in cylinder liner [45,46,47] and piston skirt [48] wear. For example, Pawlus obtained a decrease in the *Rp* parameter of 70%, but a reduction in the *Rv* parameter of 40% [47]. The *Sv* parameter can be used for detecting cracks on the surfaces.

**Figure 4:**
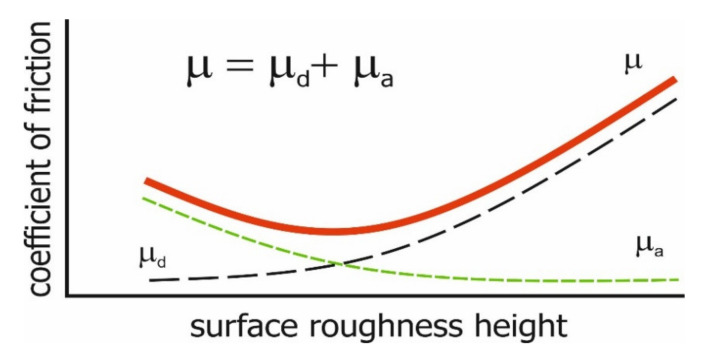
Effect of the roughness height on the coefficient of friction in dry regime.

The amplitude parameters are related to friction and wear. There are two main sources of friction: the deformation µ_d_ and adhesion µ_a_ of summits in contact (Figure 4). Under the lubrication regime, the adhesion effect is marginal; therefore, smooth surfaces frequently correspond to low friction, such as in disc-on-ball tests performed by Dzierwa et al. [49] and Sedlacek et al. [50,51]. Under dry friction, these effects can be different. The result depends on the type of contact. In the initial point contact, wear of the disc was typically higher for smaller roughness height. It is probably related to a higher maximum pressure for smoother surfaces in the initial contact point [52], which is related to the plastic deformation [53]. However, under lubricated fretting (oscillatory motion of extremely small amplitude), rough surfaces can result in smaller wear and friction compared with smooth surfaces [54,55,56]. This behaviour is caused by oil retention in surfaces of big roughness. The surfaces of marines’ shoes should be rough.

The types of friction depend on the surface height. Fluid friction occurs when oil film thickness is higher than the sum of the heights of two contacting surfaces. In the other case, mixed friction happens.

**Figure 5:**
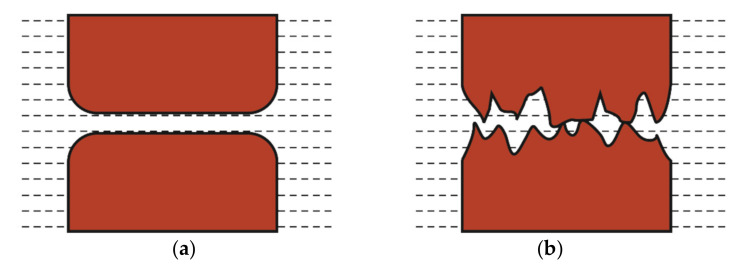
Effects of the smooth surface (**a**) and of the rough surface (**b**) on tribological performance during lubrication.

The smooth surface has an inclination to seizure due to the difficulty of maintaining oil. Stout et al. [57,58] found that a smoothly polished cylinder surface is exposed to seizure, even to more intense adhesion. Surface roughening caused an increase in seizure resistance [59]. Generally, smooth surfaces lead to seizure, and rough surfaces lead to high wear and friction (Figure 5). In lubricated sliding, smaller height corresponds to lower wear of cylinder liner [43,46] and piston skirt surfaces [48].

Higher surface roughness corresponds to a higher tendency for fatigue. The surface, which has a higher roughness, is believed to lead to a lower resistance to fatigue resistance [60,61,62,63]. The fine microstructure of additive manufactured Ti-6Al-4V seemed to positively affect the fatigue life [60]. The fatigue limit of steel specimens decreased with increasing level of roughness. The fatigue limit stress of additive manufacturing metallic parts from 316L material increased with decreasing roughness [62]. Li et al. [63] achieved a decrease in the mean fatigue life between 15 and 30% when the average surface roughness increased from 0.4 to 0.8 µm or from 0.8 to1.6 µm.

A decrease in the roughness height caused a higher corrosion resistance [15].

**Figure 6:**
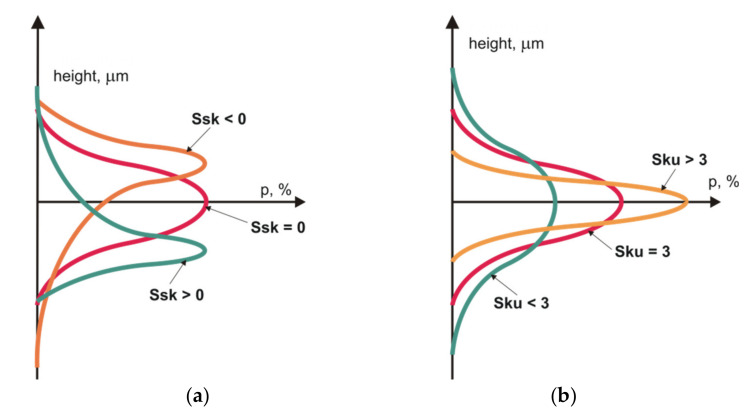
Skewness *Ssk* (**a**) and kurtosis *Sku* (**b**) of surface texture ordinate distribution.

*Ssk* and *Sku* characterise the aspect of the texture height distribution—Figure 6. The skewness is positive when the material is below the mean plane, but negative when the material is above the mean plane. Negative skewness is characteristic of multiprocess (stratified) surfaces and porous materials. These parameters were often used to identify surfaces after different machining processes; work [64] can be the example. Swirad et al. [65] found that these parameters are sensitive to burnishing parameters. Mezari et al. obtained the relation between different kinds of honing stones and skewness and kurtosis [66]. Whitehouse [67] recommended parameters characterising Beta height distribution to discriminate textures of various types; however, this proposal did not gain popularity.

**Figure 7:**
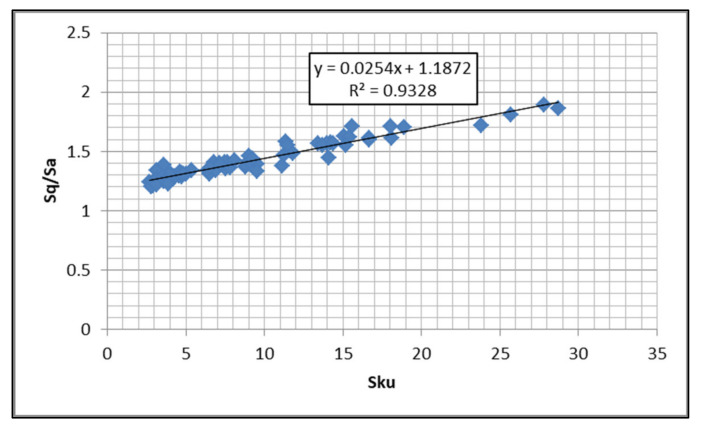
Relation between the *Sku* and *Sq/Sa* parameters of the machined surfaces.

Pawlus et al. [68,69] proposed the *Sp/Sz* and *Sq/Sa* set instead of the pair *Ssk* and *Sku*, because for highly skewed surfaces, the skewness and kurtosis are highly inversely correlated. *Sq/Sa* is particularly strongly correlated with *Sku* (Figure 7). The emptiness coefficient *Sp/Sz* smaller than 0.5 corresponds to the skewness *Ssk* smaller than 0, but *Sp/Sz* is higher than 0.5 for skewness higher than 0. Low emptiness coefficient typically leads to small wear; this finding results from theoretical analysis. [70,71].

Skewness and kurtosis are frequently used in contact studies of surfaces with non-Gaussian ordinate distribution [72,73,74,75]. A negative skewness can improve the contact of rough surfaces. Results presented in [72] show that a negative skewness and a low kurtosis improve the contact of rough surfaces by increasing the normal stiffness. Zhang and Huang [73] found that negative skewness led to an increase in the tangential stiffness. Jeng and Peng [74] revealed that skewed surfaces tended to deform more elastically. Negative skewness of smooth surfaces can improve the contact and lubrication conditions [75]. It was also found that negatively skewed surfaces of smooth [76] and rough [77] discs led to a reduction in friction and to a decrease in wear under dry sliding conditions. Gu et al. [78] found that the surface with positive skewness caused fretting crack nucleation. Chang and Jeng [75] found that skewness of —1 caused a decrease in the friction coefficient under boundary lubrication up to two times in comparison with the surface of Gaussian ordinate distribution. The tested surfaces [72,73,74,75,76,77,78] were not highly skewed.

Textured surfaces are highly skewed. Surface texturing is an option to improve the tribological performance of sliding elements. Etsion [79] and Rosenkranz et al. [80,81] published reviews in this field. Dimples (oil pockets or cavities) lead to a reduction in the frictional resistance in mixed boundary and fluid lubrications. The presence of dimples can improve the seizure resistance of sliding assemblies. Oil pockets can be also traps for abrasive particles. There are many papers on the reduction of friction and wear due to surface texturing in conditions of lubrications. References [82,83,84,85,86,87,88] are some examples of the beneficial effects of textured surfaces. Presence of dimples can enhance tribological performance of seals [82,83], journal bearings [84,85] or cylinder liners [86]. Galda et al. [87] found that textured rings could improve seizure resistance of block-on-ring contact. Surface texturing of the disc led to transition from non-conformal to conformal contact during the test in pin-on-disc configuration, which caused a decrease in the friction force [88].

Generally, negatively skewed surfaces have good lubricant retention. However, the skewness cannot characterise completely textured surfaces. Other parameters, such as pit-area ratio, dimple sizes, and oil capacity are also important. Reference [89] presents methods for the correct estimation of oil capacity. Textured surfaces are a kind of two-process texture, which contains tracks of two processes (the surface of plateau-honed cylinders is a popular example). Because the parameters *Ssk-Sku* are highly correlated, the other pair can characterise their ordinate distribution such as *Sp/Sz* and *Sq/Sa* [68,69]. For two-process textures, two parameters are proposed to describe the amplitude—they should characterise the peak and valley parts. Fecske et al. [90] recommended the *Sq* parameter and skewness for characterising texture height.

Generally, amplitude parameters are the most popular. Knowledge of other parameters is marginal. Therefore, some surfaces, which properties are related to other parameters (such as slope), are characterised by amplitude parameters.

## 3. Spatial Parameters

In contrast to the height parameters, spatial parameters are not extensions of profile parameters. These parameters should use the advantage of measuring the surface topography in three dimensions. They are based on the areal autocorrelation function. The autocorrelation function describes the correlation between a surface and this surface translated by (*tx, ty*) [11].

(5) $$f_{ACF}\left(t_{x},\;t_{y}\right)=\frac{\iint_{A}^{}z\left(x,\;y\right)z\left(x-t_{x}y-t_{y}\right)dxdy}{\iint_{A}^{}z\left(x,\;y\right)z\left(x,\;y\right)dxdy},$$

The correlation length *Sal* is the horizontal distance of the autocorrelation function at which it fastest decays to a specified value $s\in \left(0,1\right)$ [11].

(6) Sal=tx2+ty2tx, ty∈Rmin   where  R=tx,ty:fACF(tx,ty≤s,

The texture aspect ratio *Str* is the quotient of the horizontal distance of the autocorrelation function at which it fastest decays to a stated value s to the distance, at which the autocorrelation function slowed decays to s [11].

(7) Str=tx2+ty2tx, ty∈Rmintx2+ty2tx, ty∈Qmax  where  R=tx, ty:fACFtx,ty≤sQ=tx, ty:fACFtx,ty≥s   and**,

where ** is the property that the $f_{ACF\;}\geq s$ on the straight line connecting the point $\left(t_{x},\;t_{y},\right)$ to the origin.

The *Sal* and *Str* parameters contain complimentary information, therefore they both can be used for surface description. They originate from the correlation length, which is the distance at which the profile autocorrelation function decreases to a specified value. In profile analysis, this value is typically 0.1 [36], but in areal analysis it is 0.2 for practical reasons—due to the limited assessment lengths of the areal surface topography measurement, it is sometimes difficult to decay the autocorrelation function to 0.1 value. A surface with a low value of the *Sal* parameter is dominated by high frequencies in contrast to the texture characterised by a high value of the *Sal* parameter. The *Str* parameter characterises the surface isotropy; when this parameter is close to 1, the surface is isotropic, but when this parameter is close to 0, the surface is anisotropic. When the surface is isotropic, the profiles in various directions are similar to each other, contrary to anisotropic surfaces. For a strongly stationary surface, all statistical moments are time invariant [91]. In general, the parallel profiles of this surface are not substantially different from each other. The surface is ergodic when the statistical properties in various directions are the same. Ergodic surface should be stationary and stationary surfaces need not be ergodic. Any profile of the ergodic surface can be taken for the analysis of its functional behaviour. Therefore, only isotropic surfaces can be ergodic. The authors of the papers [92,93] analysed various surfaces regarding ergodicity and stationarity. Agarwal et al. [92] found that it is necessary to filter off the low frequency waves. Non-stationary surfaces are characterised by high variations of parameters [93].

**Figure 8:**
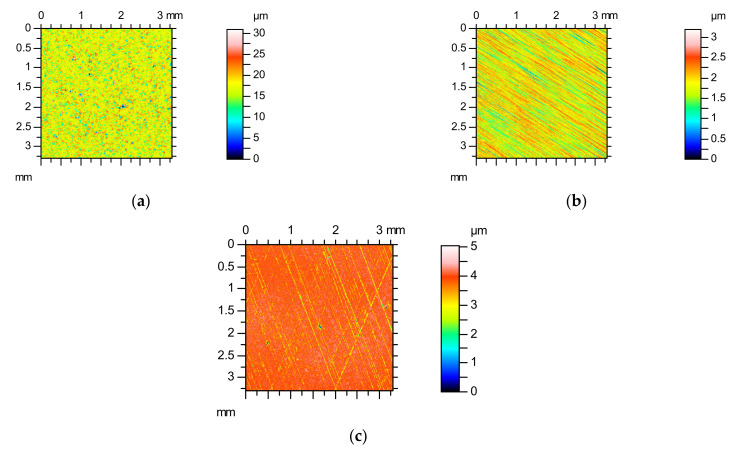
Colour-coded plots of the surface after vapour blasting (**a**), grinding (**b**), and honing (**c**).

**Figure 9:**
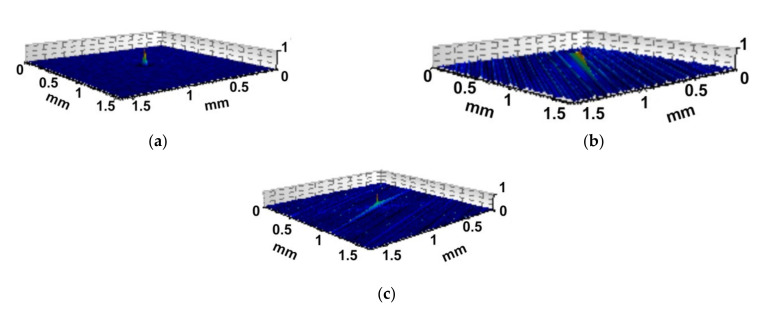
Autocorrelation functions of the surface shown in Figure 8.

**Figure 10:**
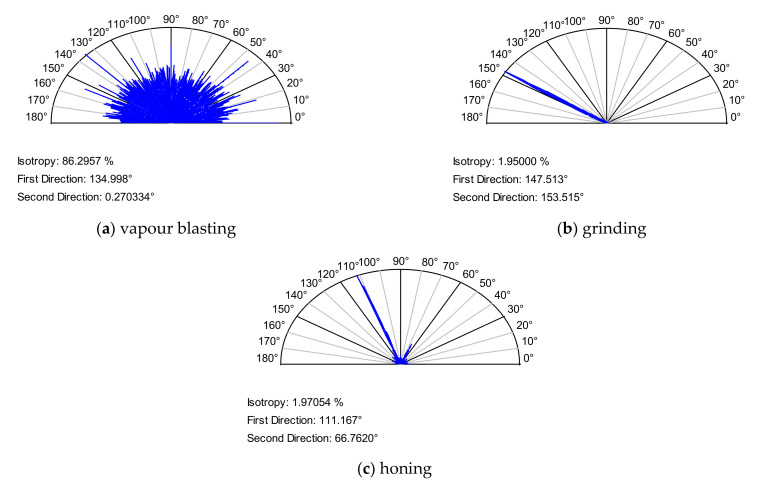
Angular spectra of the surface shown in Figure 8.

Figure 8 shows the colour-coded plots of three surfaces, Figure 9 presents their autocorrelation functions, while Figure 10 presents the angular spectra. The surface shown in Figure 8a after the vapour blasting has random isotropic character. Its roughness amplitude is high. The other surfaces presented in Figure 8 are anisotropic, of comparatively low roughness height. Ground texture shown in Figure 8b is one-directional; however, the honed surface shown in Figure 8c is cross-hatched. The autocorrelation function (Figure 9) presents additional qualitative information to the colour-coded plot about surface character (isotropic or anisotropic, random, or periodic). Fast decay of the autocorrelation function was proved on the random characters of the analysed three surfaces. Isotropic character of the vapour blasted texture is reflected by the lack of the main direction in Figure 9a. In contrast, one dominant direction is evident in Figure 9b, and two dominant directions in Figure 9c. Angular spectrum gives quantitative information about character of surfaces (isotropic or anisotropic) and about surface directionality. In Figure 10a, it is difficult to obtain the main direction. This is a characteristic feature of the isotropic surface. The isotropy presented in Figure 10 is equal to the Str parameter. This parameter does not give strict information about the character of a surface—the same value can be obtained for one-directional surfaces and cross-hatched surface. Two surfaces shown in Figure 8b,c are characterised by similar values of the *Str* parameter—near 0.02 (see Figure 10b,c). One main direction with some scatter was obtained for ground surface (Figure 10b). The honed surface has two dominant directions. The quality of the honing process can be assessed on the basis of Figure 10c. It is evident that honing grooves were not equally cut in two directions. One can obtain information on the honing angle using the angular spectrum.

The angular spectra shown in Figure 10 were obtained on the basis of the power spectral density functions [94]. Other methods can also be used to obtain the surface directionality plot, such as the cross-correlation function [95,96] or the autocorrelation function [97,98]. Pawlus et al. [99] developed a special method for characterising the directionality of honed cylinder surfaces on the basis of the dimensions of deep grooves.

Biboulet et al. [100] found that the cross-hatched cylinder liner texture provides load-carrying capacity. Valleys perpendicular to the sliding direction generate the highest load-carrying capacity. For smooth plateau parts, larger spacing between valleys led to an increase in load-carrying capacity [101]. Pawlus [102] revealed that cylinder liner abrasive wear was higher for larger distance between deep valleys. A decrease in axial distance between deep valleys from 500 to 200 µm led to a decrease in liner wear of about 30%. Because the honing angle and the spacing between honing grooves are functionally important parameters [100,101,102], a comprehensive description of the spatial properties of the cross-hatched structures is needed. Classical spatial parameters do not also describe the pattern of textured surfaces [103], although a method of their description was developed [104].

**Figure 11:**
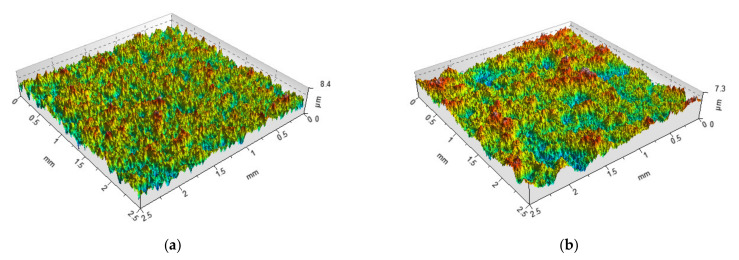
Isometric views of textures modeled by authors described by the *Sq* parameter of 1 µm and correlation length of 50 (**a**) and 100 µm (**b**).

Horizontal parameters were applied for the characterisation of the surface profile. Whitehouse [36] used the correlation length. The random Gaussian profile can be described by the *Rq* parameter and correlation length. According to Whitehouse [36], the random profile has an exponential shape of the autocorrelation function. The profile shape can be described by three initial points of non-normalised autocorrelation function [105]. The non-Gaussian random profile is characterised by the correlation length, and parameters *Rq, Rsk,* *Rku* [106]. Therefore, random profiles can be modelled on the basis of those parameters. Modelled areal (3D) Gaussian surface is characterised by the *Sq* parameter and correlation lengths in orthogonal directions [24,25,26]—Figure 11. This method can be used for the simulation of the isotropic surface and anisotropic one-directional surface. However, modelling the crossed surface is more difficult [107,108]. This surface is also characterised by correlation lengths. Areal surface of the ordinate distribution different from the Gaussian surface is characterised by the *Sq, Ssk*, and *Sku* parameters and correlation lengths in perpendicular directions; therefore, these parameters are input values during surface simulation [25,109,110,111]. Hu and Tonder [25], Wu [109], and Wang et al. [110,111] used the Johnson translation system in surface modelling. This method is not good for generation of two-process surfaces. The imposition method is better. However, two-process surface is also characterised by correlation lengths in perpendicular directions [108,112].

Typically, a larger correlation length corresponds to better functional properties [113,114]. Hirst and Hollander [113] found that the load-carrying capacity in boundary friction is higher with higher correlation length. A larger correlation length [114] in the sliding direction corresponds to a smaller wear during lubrication. Whitehouse and Archard [36] found that short wavelengths were removed during the running process, in contrast to long wavelengths. Prajapati et al. [115] revealed that the correlation length *Sal* increased during running-in. The comfort of passengers in a car is higher for higher wavelengths of the road [16]. However, the large separation between grooves led to high stress concentrations, and hence to a reduction in fatigue life [116].

The condition of contact between rough surfaces depends on the ratio of anisotropy [117]. The plasticity index of surface texture is related to the anisotropy ratio of anisotropy [118]. The coefficient of friction in cold rolling depends on the ratio of anisotropy of the sheets [119].

**Figure 12:**
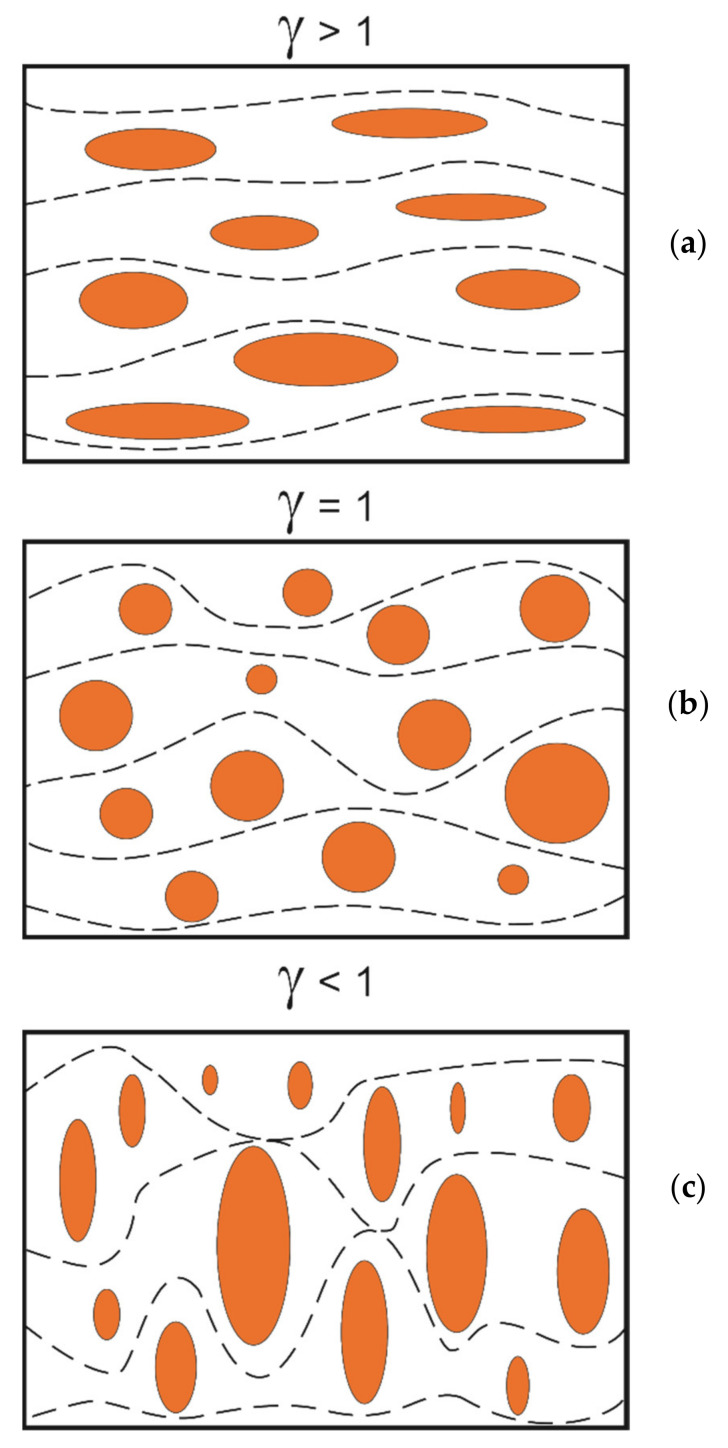
Orientations of rough surface to the movement directions, after [120]. (**a**) surfaces oriented longitudinally, (**b**) isotropic surfaces, (**c**) surfaces oriented transversely.

The position of the one-directional surface with respect to the sliding direction is important in lubrication. Patir and Cheng [120] developed one of the first models of the oil flow. They analysed the surfaces of various anisotropy ratio γ, which is the ratio of the correlation lengths in orthogonal directions (Figure 12)—this ratio was first developed by Kubo and Peklenik [97]. Surfaces oriented longitudinally in partial hydrodynamic lubrication (γ > 1) do not cause pressure resistance, only a small side flow is permitted; for isotropic surfaces (γ = 1), the main and side flows are similar. Surfaces oriented transversely (γ < 1) led to increases in main flow resistance and to the addition of the side flow.

Generally, the transverse orientation leads to a decrease in the friction coefficient and to an increase in load capacity. This finding was confirmed in recent research in mixed-EHL (elastohydrodynamic) lubrication of rough surfaces [121,122] and in HL (hydrodynamic) lubrication [123].

The transverse orientation of the roughness typically improves the tribological parameters in mixed and boundary friction. Moronuki and Furukawa [124] found that the friction reduction under low pressure was greater when the valleys were orthogonal to the sliding direction compared with grooves parallel to the sliding direction and smooth surfaces. Petterson and Jacobson [125] found that the transverse grooves offered low friction under boundary lubrication, in contrast to longitudinal ones. Yuan et al. [126] discovered that when pressure was low, the friction reduction effect of grooves orthogonal to the direction of motion was higher than that of parallel grooves. Similar effects were obtained by Zum-Gahr et al. [127]. However, different effects of groove orientation can be obtained for other operating parameters, such as the contact pressure. The authors of papers [128,129,130,131,132] found that ellipsoidal dimples should be positioned perpendicularly to the sliding direction to improve tribological properties. Qiu et al. [128] obtained the highest load-carrying capacity under gas-lubricated sliding for the ellipsoidal oil pockets oriented orthogonally to the direction of motion. Vladescu et al. [129] found that the best tribological performance under mixed and boundary lubrications under the reciprocating motion was achieved for grooves positioned perpendicularly to the sliding direction. Lu and Khonsari [130] obtained better tribological performance of elliptical dimples over circular ones under mixed lubrication. Oval dimples oriented perpendicularly to the sliding direction can lead to a decrease in the coefficient of friction in starved lubrication under reciprocating motion; however, the results depend on the ratio of the major to the minor axes of the ellipse [131]. The elliptical dimples showed the friction reduction up to 30%; compared with behaviour of untextured specimens in reciprocating motion, squared and circular oil pockets offered a smaller reduction [132].

The honing angle is also important. It is typically between 45 and 55 degrees. There are opinions that the smaller honing angle led to friction reduction. For example, Bolander and Sadeghi revealed after numerical modelling that for higher honing, angle friction would be higher [133]. Michaill and Barber [134] found that the low honing angle enhanced hydrodynamic lift. Jocsak et al. [135] thought after numerical simulation that a reduction in the honing angle caused a lower friction of the piston ring–cylinder system. The simulation performed by Spencer et al. [136] revealed that a lower honing angle ranging from 25° to 75° produced higher oil film thickness. The highest increase (from 5.11 to 5.127 µm) was achieved for the honing angle of 35°. Grabon et al. [137] found after experimental investigation that a honing angle smaller than 55 degrees caused smaller coefficient of friction than higher angles. Directionality plots of the honed cylinder surface can be used for quality inspection of the machining process.

**Figure 13:**
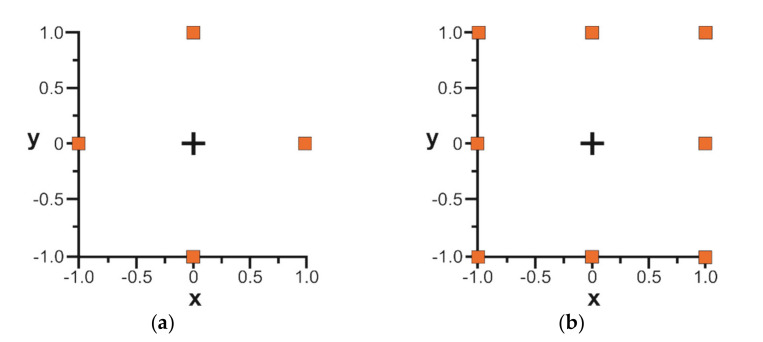
Various methods of identification of summits, on the basis of four (**a**) and eight neighbouring points (**b**).

In the older standard [9] the density of summits *Sds* was the spatial parameter. It is the number of summits in a sampling area. There are problems with the definition of summit. It is a point which the ordinate is higher than those of neighbouring points. However, the question arises: how many neighbouring points should be analysed? Summit can be identified on the basis of four, eight [138,139]—Figure 13—or six neighbouring points [140]. Summits can be also identified in the autocorrelation area [93]. From comparison of the number of summits calculated using spectral moments [5] and obtained from surface topography, definition of summits on the basis of eight measuring points was recommended [137,138] and was used in many research works. However, this method is sensitive to the sampling interval. Smaller summits can be a part of a bigger summit. Researchers developed some procedures to obtain a stable value of the density [141]. For this reason, the density of summit *Spd* was transferred to the peak density in standard ISO 25178-2. The *Spd* parameter takes into account only those significant summits that remain on the surface after a discrimination by segmentation [142]. However, this approach originated from geomorphology and the classical definition of summit is used in contact mechanics. The density of summits is an important parameter in contact mechanics, especially when statistical contact models are used. Recently, it was found that one can predict the density of summits *Sds* parameter (in contrast to the *Spd* parameter) of an equivalent sum rough surface when the summit density of surfaces in contact are known [69]. The main texture direction *Std* was previously a spatial parameter in older standard [9]. This parameter exists in the new ISO 25178-2 norm as a miscellaneous parameter. It depends on the surface orientation during a measurement.

## 4. Hybrid Parameters

ISO 25178-2 standard contains only two hybrid parameters. The rms. slope (surface gradient) *Sdq* is calculated using the following equation [11]:

(8) $$Sdq=\sqrt{\frac{1}{A}\iint_{A}^{}\left[{\left(\frac{\partial z\left(x,y\right)}{\partial x}\right)}^{2}+{\left(\frac{\partial z\left(x,y\right)}{\partial y}\right)}^{2}\right]dxdy},$$

The developed interfacial areal ratio *Sdr* is the ratio of the increment of the interfacial area [11].

(9) $$Sdr=\frac{1}{A}\left[\iint_{A}^{}\left(\sqrt{\left[1+{\left(\frac{\partial z\left(x,y\right)}{\partial x}\right)}^{2}+{\left(\frac{\partial z\left(x,y\right)}{\partial y}\right)}^{2}\right]-1}\right)dxdy\right],$$

**Figure 14:**
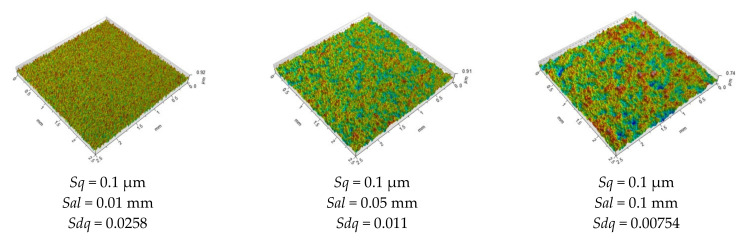

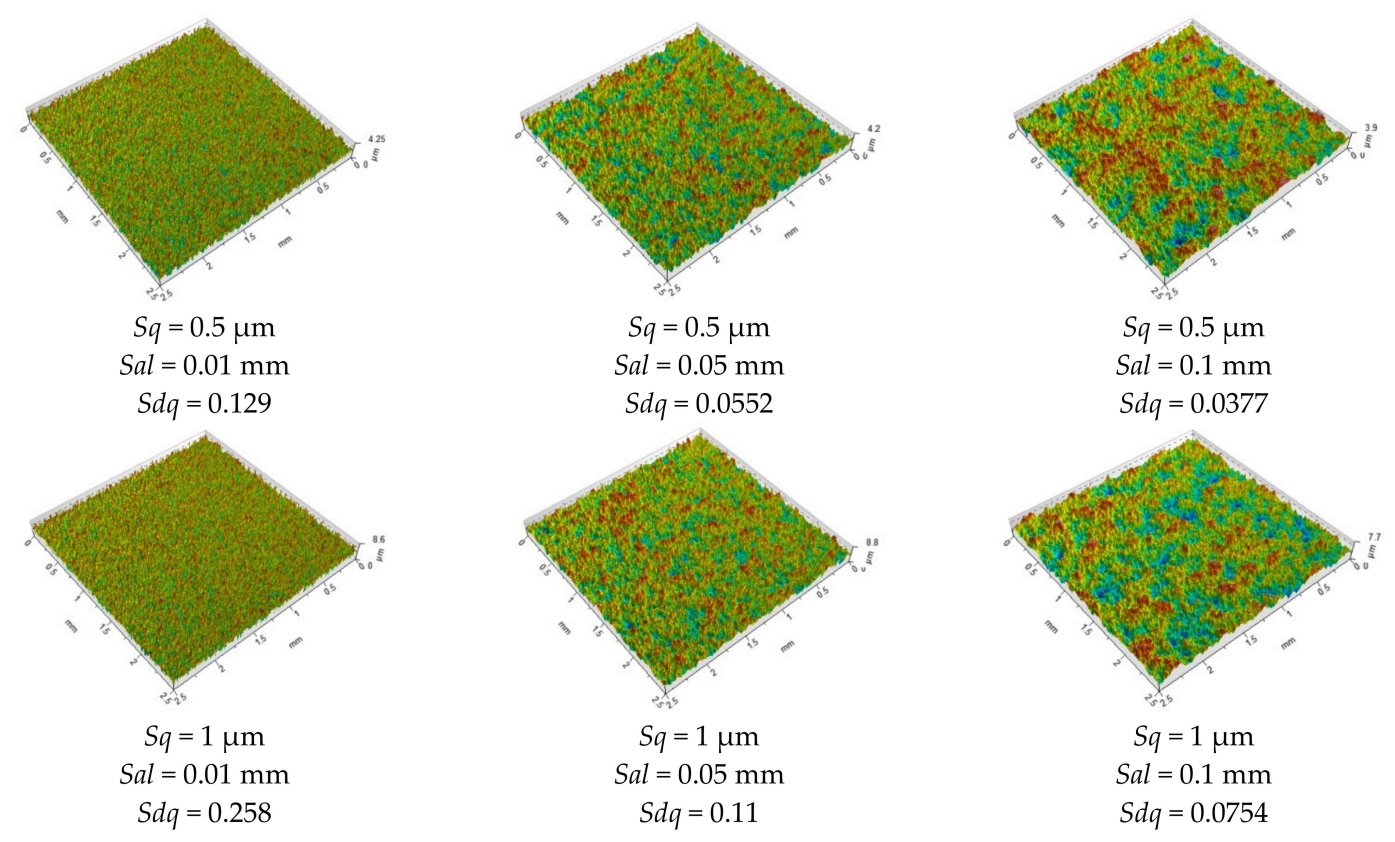
Modeled by authors Gaussian random surfaces with the parameters charactering them (description in text).

The hybrid parameter combines information on height and spatial parameters. Therefore, one parameter can contain information on each surface. These parameters are higher for bigger surface amplitude and for smaller main surface wavelength. Figure 14 presents isometric views of the modeled isotropic surfaces of the Gaussian ordinate distribution. They are characterised by height described by the *Sq* parameter (0.1, 0.5 and 1 µm) and wavelength characterised by the correlation length *Sal* (0.01, 0.05 and 0.1 mm). One can see that an increase in the *Sq* parameter caused an increase in the slope *Sdq*. When the height increased 10 times, *Sdq* also increased 10 times. An increase in the correlation length *Sal* caused a decrease in the slope *Sdq*. However, when the correlation length increased 10 times, the *Sdq* parameter decreased only 3.4 times. In reality, typically the slope of random surfaces is more correlated with amplitude than surface wavelength.

These *Sdq* and *Sdr* parameters are interrelated. The *Sdr* parameter can be approximated on the basis of the *Sdq* parameter:

(10) $$Sdr\;=\;Sdq^{2}/2,$$

For the sampling interval of 3. µm and comparatively smooth surfaces (*Sq* < 1 µm), the errors of *Sdr* parameter determination were smaller than a few percentages. For the *Sq* parameter smaller than 0.5 µm, errors were typically smaller than 1 percent. Large errors occurred for extremely rough surfaces. However, increasing the sampling interval caused a reduction in errors in the prediction of the *Sdq* parameter using formula (10). For comparatively smooth surfaces measured with reasonable sampling intervals, only one hybrid parameter is recommended for surface description.

Because the *Sdq* and *Sdr* parameters are interconnected, the correlation between them is extremely high (for example, Czifra and Baranyi [143] obtained the determination coefficient of 0.998).

The second spectral moment of the surface profile is the square of profile rms. slope. Therefore, the *Sdq* value of the equivalent surface can be precisely predicted on the basis of rms. slopes of each surface in contact [69]. Rms. slope can be used to evaluate surface anisotropy [144]. For example, the ratio of the slopes in two perpendicular directions depends on the honing angle, therefore it can be used to describe cylinder anisotropy. To obtain a local slope, formulas based on 2, 3, and 7 neighbouring points were used. Computation of surface slope on the basis of 2-and 7-point formulas was recommended [145,146]. There are problems with measurement surfaces of high slopes typically using optical methods [147,148,149]. As the result of high slopes presence, the surface points cannot be detected. Sharp edges cause the presence of outliers, called spikes—they are high and narrow peaks (of Dirac type) that did not really exist on the surface. Lu et al. [147] developed an optical sensor based on the focus detection method. Deviations from the step heights were obtained due to no light signals being reflected. The slope is limited, especially in surface texture measurement using interferometers [148]. The result from an interferometric measurement presented optically introduced artifacts due to the presence of high local surface slopes [149]. Some surface texture measuring instruments measure slope directly.

Hybrid parameters are super sensitive to high frequency noise and often hardly comparable between different instruments.

Surface slope is related to friction, wear, light reflection, hydrodynamics, and spalling [150]. Slope is not an intrinsic texture property; therefore, various scales of slope should be analysed. Torrance [151] found the relation among surfaces slopes at various scales and the boundary friction and wear of cam rocket pairs. The coefficient of friction depends on the slope of asperities of the harder surface. Elvasli et al. [152] obtained a substantial effect of surface slope on wear in dry and lubricated reciprocating sliding. Berglund et al. [153] achieved a strong linear correlation (0.83) between the *Sdq* parameter of milled die steel surface and the coefficient of friction. An increase in the *Sdq* parameter from 0.2 to 0.3 caused an increase in the friction coefficient from 0.13 to 0.16. Childs [154] found that surfaces with low slope tended towards elastic contact. Typically, friction is proportional to the surface slope. A high slope corresponds to high surface ability for plastic deformation. Some version of the plasticity index depends on the slope [38,155,156]. For a larger slope, the tendency for plastic deformation is higher. The *Sdq* parameter is useful in sealing applications and for controlling the cosmetic appearance of surfaces [157].

Pagani et al. [158,159] proposed a modification of the *Sdr* parameter. For example, the parameter defined in [159] was used to characterise re-entrant features, which increase the specific surface area in additive manufacturing.

When the *Sdr* parameter is higher, the surface ability to adhesive joints creation is also higher [160,161,162]. Zielecki et al. [160] found that the shear strength of the S235JR steel lap adhesive joints was strongly linearly (0.74) correlated with the *Sdr* parameter. An increase in the *Sdr* parameter from 0.2 to 9% caused an increase in the shear strength from 10 to 20 MPa. Van Dam [161] obtained higher average ultimate shear strength from single-lap joint specimens of a steel-epoxy adhesive interface for higher values of the *Sdr* parameter. Zheng et al. [162] found that the lap-shear joint of adhesive-bonded magnesium AZ31B was proportional to the actual surface area. The developed surface area is related to coating adhesion and corrosion protection [163]. Because roughness height is frequently proportional to the interfacial area, the ability of adhesive joints creation was found to be sometimes proportional to surface amplitude [161]. This finding is also a consequence of bad or/and misunderstood knowledge about non-height parameters. Blunt and Jiang [164] found that the *Sdr* parameter of an in vivo femoral stem decreased as a result of abrasive wear in vivo.

The older proposal [9] also contained the mean summit curvature *Ssc* as the hybrid parameter. This parameter is related to the m4 spectral moment; therefore, it can be predicted for the equivalent sum surface when the values of this parameter of both surfaces in contact are known [69]. However, the accuracy of the prediction of this parameter is worse compared with that of parameters *Sq* and *Sdq.* The reciprocal of the parameter *Ssc* is the mean radius of summits, frequently used in contact mechanics. This parameter is included in the plasticity index [165,166]. When the mean summit radius is higher, the tendency for plastic deformation, and hence, wear, is lower. For random surfaces, the *Ssc* parameter is highly correlated with the *Sdq* and *Sdr* parameters. The parameter *Ssc* was replaced by the Spc parameter in ISO 25178-2 standard. Similar to *Spd*, the *Spc* parameter takes into consideration only significant summits [142]; therefore, this parameter belongs to the group of feature parameters.

## 5. Other Field Parameters

**Figure 15:**
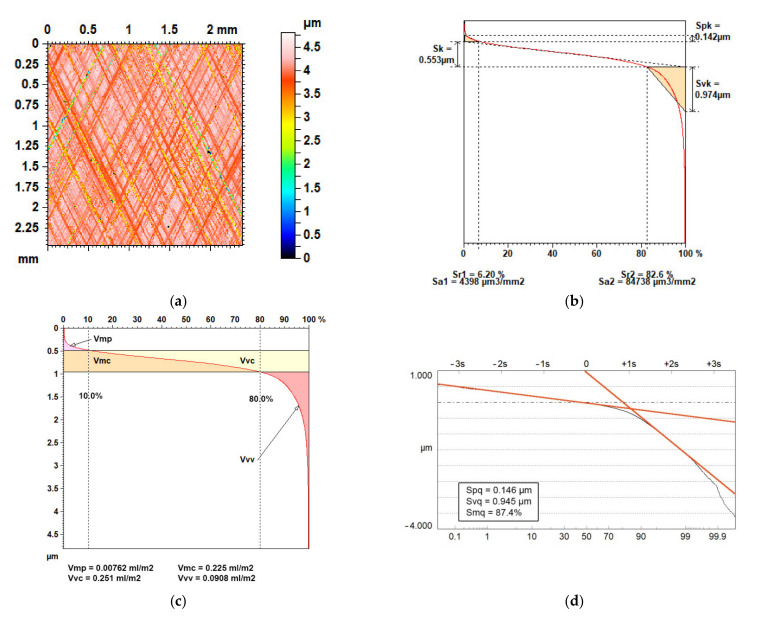
Colour-coded plot of plateau-honed surface (**a**), material ratio curve with parameters from *Sk* group (**b**), *V* group (**c**), and *Sq* group (**d**).

Some of the remaining parameters are related to the areal material ratio curve. They are called functional parameters. There are three families of parameters: the *Sk* group, the *V* group, and the *Sq* group. They are presented in Figure 15 for the same plateau-honed surface. The following parameters belong to the *Sk* family: the core height *Sk*, the reduced peak height *Spk*, the reduced dale height *Svk*, and *Sr1* and *Sr2* material ratios. The *V* group consists of the following parameters: the dale void volume *Vvv*, the core void volume *Vvc*, the peak material volume *Vmp*, and the core material volume *Vmc*. The default material ratios used to calculate these parameters are 10 and 80%. There are three parameters of the *Sq* family: the plateau rms. deviation *Spq*, the dale rms. deviation *Svq*, and the material ratio at the transition point between the valley and plateau regions *Smq*. There are also similar parameters that describe profiles from the *Rk* and *Rq* groups.

Parameters from the *Sq* group can be applied only to two-process surfaces, in contrast to other parameters. They are obtained from the probability plot of the material ratio curve—Figure 15d. For the one-process surface of random character, one straight line is visible (see Figure 2), while for the texture of two processes, there are two straight lines (Figure 15d). The idea of the *Sq* group is to divide the surface into two parts: peak and valley (dale). In contrast, the idea of the *V* and *Sk* groups is to divide the surface texture into three parts: peak, core, and valley. Division of the surface texture into two parts is based on the two-process character of the surface (surface is created in two processes). Therefore, *Sq* parameters can be used in two-process surface modelling [27]. However, the division of texture into three parts seems to be correct from a surface functioning point of view. It is supposed that the peak part is responsible for the running-in, the core for steady state, and the valley for problems related to the lack of lubricant. However, the evidence is lacking. Parameters from the *Sk* group are easy to calculate; the calculation of parameters from the *V* group is also comparatively easy. However, incorrect application of the *Sk* parameters can lead to serious errors. The probability of errors in calculating parameters from the *V* group is lower. However, they are based on an arbitrary assumption (default material ratios). Combining *V* and *Sk* methods is a good idea [167]. In contrast, the calculation of the parameters from the *Sq* group is difficult. However, these parameters have a strong theoretical background. The parameters from these three groups or were developed for plateau-honed cylinder surface [168,169,170,171]. They were critically reviewed and compared in [172]. Nielsen [168] presented usefulness of parameters from *Rk* family for evaluation of sintered, honed, and ceramic bearing surfaces. Zipin [169] thought that the *Rq* group of parameters is superior to the *Rk* family. Raja et al. [170,171] underlined the advantages of the *Rk* family of parameters for characterising plateau-honed surface texture. The *Sk/Rk* group is the most frequently used. The oil consumption, and hence exhaust emission, is higher for bigger values of the *Sk* parameter [173]. Therefore, this parameter is minimised, in contrast to the *Svk* parameter related to the oil capacity [174].

**Figure 16:**
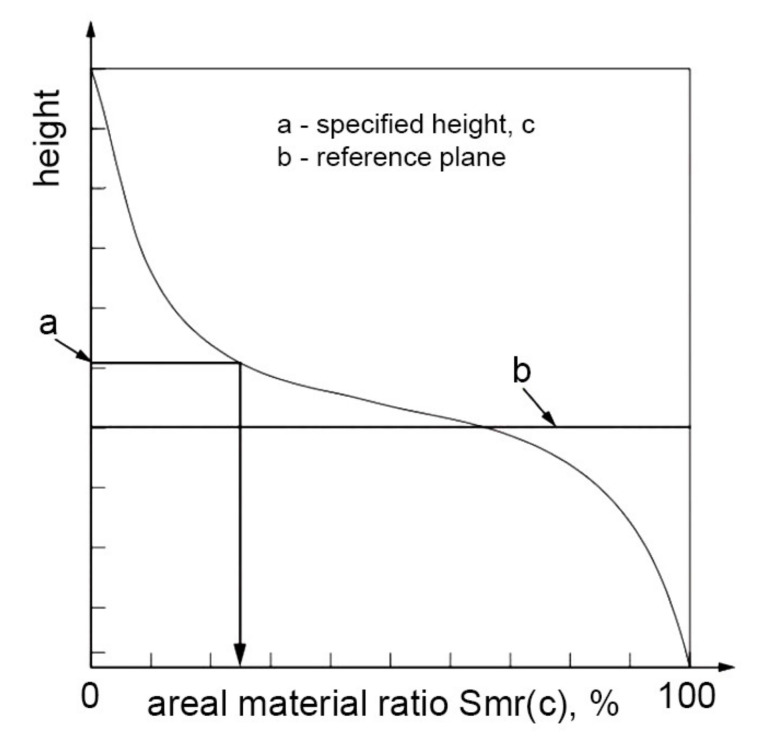
Interpretation of the *Smr* parameter, after [11].

There are also other parameters related to the material ratio curve: areal material ratio *Smr*, inverse material ratio *Smc*, and extreme height of the peak *Sxp* [11]. The *Smr* (c) parameter is the ratio of the area of the material of the specified height c to the assessment area. The height is taken from the reference plane (see Figure 16).

**Figure 17:**
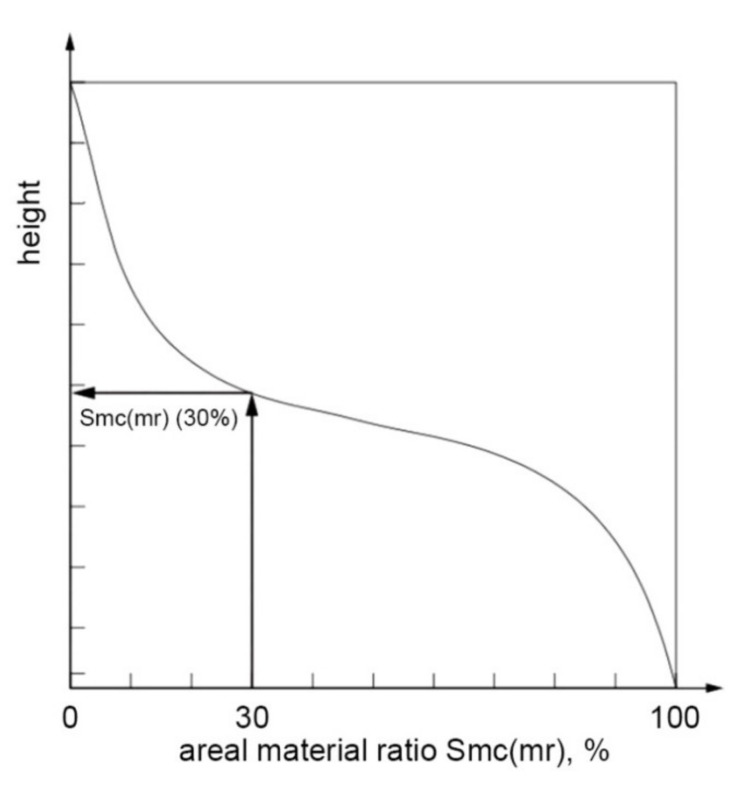
Interpretation of the *Smc* parameter, after [11].

*Smc* (mr) is the height corresponding to a given material ratio (mr) [11]—see Figure 17. Commercial software calculated the Smr parameter on the basis of the highest point (peak) of the surface.

Sxp is the difference between material ratios p and q [11].

Material ratio curve has various useful applications such as determination of oil capacity, pit-area ratio of textured surface, or a low wear assessment. It can be used not only for cylinder liner surfaces, but also for other textures, for example, after additive manufacturing [175].

There is also a group of fractal parameters, defined by Brown [176,177,178] in ISO 25178-2 -standard. This method, called the patchwork method, uses triangular patches to estimate the surface area as a function of the patch area. There are also different methods for calculating fractal parameters. The fractal dimension of the areal surface is higher than 2 and smaller than 3 [179]. Fractal analysis of surfaces started from the publication of the paper [180] written by Sayles and Thomas. The problem is that fractal parameters can be used only for fractal surfaces. Fractal surfaces are continuous but not differentiable. Self-similar surfaces look the same for various scales of sizes. The properties of self-affine textures are more restricted. There are different opinions on the fractal character of surfaces. For example, Majumdar and Bhushan thought that surfaces created by random techniques are fractal, while surfaces created by a deterministic technique are non-fractal [181]. In contrast, Whitehouse [182] thinks that surfaces created by random manufacturing processes are non-fractal but Markov. Fractal areal surface textures and profiles can be modeled [183]. Fractal surfaces are applicable in various areas, such as contact mechanics [184,185,186,187] and wear [188,189,190,191]. Many authors of paper [184] solved problem on the adhesion between nominally flat fractal surfaces. Hanaor et al. [185] calculated interfacial stiffness and contact area evolution between two rough self-affine fractal surfaces. In [186], they analysed asperity interactions of contacting fractal surfaces. Paper [187] presents a fractal contact model for nominally flat rough surfaces maintaining the concept of surface asperities. Zhou et al. [188,189] predicted the wear rate in terms of two fractal parameters. Rosen et al. [190] analysed changes in fractal parameters of plateau-honed cylinder surfaces during wear. Shirong and Gouan [191] developed a fractal model of wear during the running-in of self-affine surfaces.

In ISO 25178-2 standard there is also one miscellaneous parameter—texture direction Std. The value of this parameter depends on the location of surface to the measurement directions. Its usefulness is restricted. It can be applied to relocate surfaces during machining or wear. It can be a reference during changing the position of the surface during measurement [192].

## 6. Feature Parameters

In the calculation of field parameters, every surface point is considered. Feature parameters are defined from a subset of topographic features. The feature parameters take into consideration only recognised surface features. Therefore, they can be used only in special situations. The feature parameters originated from geography and cartography.

**Figure 18:**
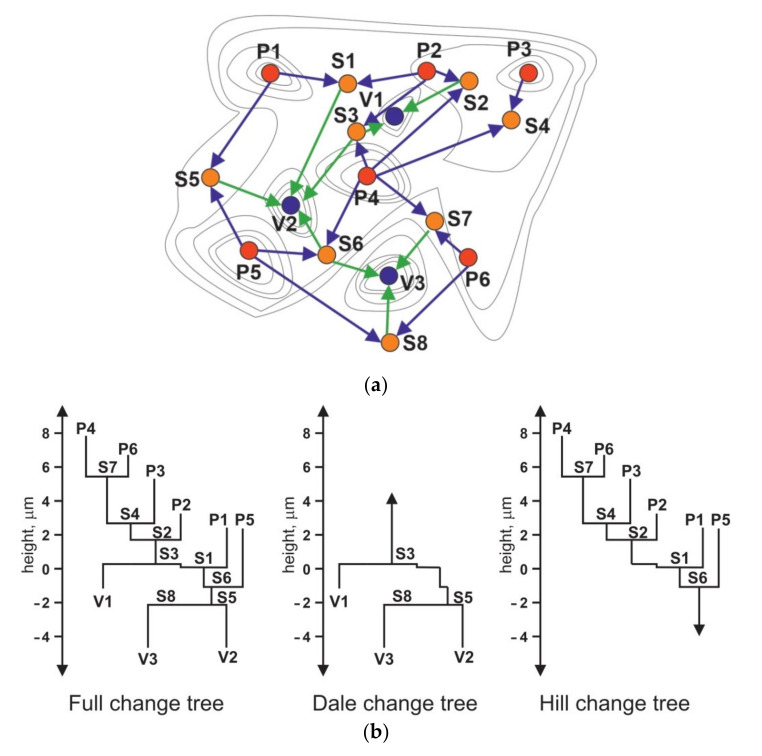
Contour map with critical points and lines (**a**), change tree with peaks (P), pits (V), and saddle points (S) (**b**), after [11].

There are the following stages of feature characterisation: selection of the type of feature, segmentation, determining significant features, selection of attributes of the feature, and then quantifications of feature statistics. There are areal (hills, dales), line (course line and ridge line), and point features (peaks, pits and saddle points). Dales and hills are equivalents of profile motifs. A hill is a region around a peak (local maximum), while a dale is a region around a pit (local minimum). In watershed segmentation, virtual water was poured over the surface. As a result, all dales were segmented by ridge lines. Figure 18 a shows contour plot with critical points and lines. Peaks P1–P6, saddle points S1–S8, and pits V1–V3 are critical surface points connected by ridge lines and saddle lines. The hill change tree characterises the connection between peaks and saddle points, while the dale change tree describes the relations between pits and saddle points. The full change tree represents the relationship between critical points in the hills and dales (Figure 18b). Peak and pits represent termination of lines, while saddle points are represented by merging of two or more lines into one line.

Smaller segments are then pruned out (Wolf pruning), to avoid over-segmentation [142,193]. Wolf pruning is done typically using a percentage of a total surface height (typically 5% of *Sz*). Only significant features are used in the characterisation. The calculation procedure is difficult.

There are the following feature parameters [11]:

- Density of peaks *Spd*;
- Arithmetical average peak curvature *Spc*.
- These parameters replaced the *Sds* and *Ssc* parameters of the older standard proposal [9].
- There are also [11]:
- Ten-point height *S10z*;
- Five-point peak height *S5p*;
- Five-point pit height *S5v*;
- Average dale area *Sda*;
- Average hill area *Sha*;
- Average dale volume *Sdv*;
- Average hill volume *Shv*.

The feature-based characteristion technique was used in various areas, such as tribology [194,195], machining [196,197], and biomedicine [198]. Hao et al. [194] applied it to the analysis of disc and ball surfaces after tribological tests. Tian et al. [195] used feature parameters to assess the surface topography of the wear particle. Ye et al. [196] applied the feature-based characterisation technique to characterise the topography of the diamond grinding wheel. Feature characterisation was used for surfaces of electroplated diamond tools [197]. Wang et al. [198] characterised biomedical titanium surfaces by feature parameters. The watershed segmentation method is also suitable for analysis of additively manufactured freeform surfaces [199]. Other applications are presented in reviews [200,201,202]. However, Zabala et al. [203] found a limited ability of the *Spc* parameter to characterise dental implant surfaces.

## 7. Functional Importance of Parameters

To select parameters from various groups, information on the functional significance of the parameters is substantial. It is listed in Table 1. Fractal parameters and parameters from the *Sq* family (*Spq,* *Svq*, and *Smq*) were not taken into consideration because they can be used only for special types of surfaces. Feature parameters were not presented too, since they can be used only for specific applications. The Std parameter of low functional significance was also not analysed. Not only *Sa, Sq, Sz, Sp*, and *Sv* parameters characterise surface amplitude. Similar information can be obtained on the basis of parameters from the *V* group, *Sk,* *Spk*, and *Svk*. In Table 1, only the parameters from the ISO 25178-2 standard [11] are presented. Therefore, *Ssc* and *Sds* are not included. These parameters are important in contact mechanics of rough surfaces. Real areas of contact and contact load depend on the mean radius of curvature of summits, which is the reciprocal of the *Ssc* parameter, and on the density of summits *Sds*. The possibility of plastic deformation is larger for higher values of the Ssc parameter. The total contact area and contact load are obtained by summing the individual asperity contributions; therefore, they are proportional to the Sds parameter in statistical contact models.

From the study of Table 1, we can conclude that most of the parameters are related to friction and wear. However, the selection of parameters should be dedicated for special applications. Only the most important relations are listed in Table 1. Detailed information about the functional significance of the parameters was given in Section 2, Section 3, Section 4 and Section 5.

## 8. Conclusions and Outlook

This review presents definitions of areal surface texture parameters, especially included in the ISO 25178-2 standard [11]. Functional importance of these parameters is discussed.

Surface texture parameters can be selected on the basis of various criteria. Definitions of the parameters should be known. The parameters should be easy to calculate. They should be function-relevant, having low sensitivity to the measurement errors. The number of parameters should be small. Parameters characterising surface textures should belong to various groups (height, spatial, hybrid, and others). Selected parameters ought to be statistically independent. A higher number of parameters should characterise multi-processes than one-process textures.

Because the spatial parameters *Sal* and *Str* are complimentary, both can characterise the spatial surface properties. However, they cannot characterise completely some surface types, such as cross-hatched textures of honed cylinder liners. For textures measured with reasonable sampling interval, only one hybrid parameter from the *Sdq–Sdr* set is required for surface description; the *Sdq* parameter is preferred. The feature parameters, difficult to calculate, should be used only in special applications.

Amplitude parameters are the most frequently used. Knowledge of other parameters is marginal. The amplitude parameters are related to friction, lubrication, and wear. They are used for technical control of manufacturing. The *Sq* parameter is useful in the study of surface deformation of surfaces and in surface modelling. Interpretation of the *Sv* and *Sp* parameters is important. The *Sv* parameter characterises the material volume, while the *Sp* parameter describes the void volume of the surface texture.

Skewness *Ssk* and kurtosis *Sku* characterise the shape of the height distribution. A negative skewness typically improves the contact of rough surfaces; it leads to friction reduction and good lubricant retention. Because the parameters *Ssk–Sku* of two-process textures are highly interrelated, the other pair is proposed to characterise the shape of height distribution: *Sp/Sz* and *Sq/Sa*—it can be also used for the description of one-process textures.

Orientation of an anisotropic one-directional surface to the sliding direction is tribologically important. The transverse orientation of the asperities typically leads to better tribological properties as compared with the longitudinal position. Directionality plots of cross-hatched cylinder textures are helpful in their quality inspection. Spatial parameters *Sal* and *Str* are useful in Gaussian surface modelling.

Hybrid parameters are related to contact between rough surfaces, friction, wear, sealing, and cosmetic appearance. When they are higher, the surface ability to create adhesive joints increases.

The parameters connected with the material ratio curve are related to friction and wear. This curve has various useful applications such as determination of oil capacity, determination of pit-area ratio of textured surface, or a low wear assessment. It is used in technical control of honed cylinder surfaces. The main problem is the selection of groups of parameters that describe the material ratio curve.

Looking into the future, the authors think that the tendency to search for new functionally important parameters for will be continued. For example, in a new version of this ISO standard that will be released soon, a new parameter—dominant spatial wavelength (*Ssw*)—will appear, as there is a need for that. The idea to look for a single parameter for a particular application is still actual. Feature-based characterisation is still in development and more research efforts are needed. The feature parameters should be configured by the user and adjusted to needs. Fractal parameters should be modified.

## Figures and Tables

**Table 1:** Functional importance of groups of areal parameters.

Type of Parameters	Examples	Functional Importance
Amplitude	*Sa, Sq, Sz, Sp, Sv*	Surface contact, lubrication, friction, wear, fatigue, technical control of manufacturing
Characterising the shape of the height distribution	*Ssk, Sku, Sp/Sz, Sq/Sa*	Surface contact, friction, wear
Spatial	*Sal, Str*	Lubrication, friction
Hybrid	*Sdq, Sdr*	Surface contact, friction, wear, ability to adhesive junctions, sealing, and cosmetic appearance
Related to material ratio curve	*Sk, Spk, Svk, Sr1, Sr2,* *Spq, Svk, Smq, Vvv,* *Vmp, Vmc, Vvc, Smc,* *Smr, Sxp*	Wear, friction, oil capacity, low wear assessment, technical control of manufacturing

## Data Availability

Not applicable.
